# Redating the earliest evidence of the mid-Holocene relative sea-level highstand in Australia and implications for global sea-level rise

**DOI:** 10.1371/journal.pone.0218430

**Published:** 2019-07-17

**Authors:** Amy J. Dougherty, Zoë A. Thomas, Christopher Fogwill, Alan Hogg, Jonathan Palmer, Eleanor Rainsley, Alan N. Williams, Sean Ulm, Kerrylee Rogers, Brian G. Jones, Chris Turney

**Affiliations:** 1 School of Earth, Atmospheric and Life Sciences, University of Wollongong, Wollongong, New South Wales, Australia; 2 Palaeontology, Geobiology and Earth Archives Research Centre, and ARC Centre of Excellence for Australian Biodiversity and Heritage, School of Biological, Earth and Environmental Sciences, University of New South Wales, New South Wales, Australia; 3 School of Geography, Geology and the Environment, Keele University, Staffordshire, United Kingdom; 4 Waikato Radiocarbon Laboratory, University of Waikato, Hamilton, New Zealand; 5 Extent Heritage Pty Ltd, Pyrmont, New South Wales, Australia; 6 ARC Centre of Excellence in Australian Biodiversity and Heritage, College of Arts, Society and Education, James Cook University, Cairns, Queensland, Australia; Technische Universiteit Delft, NETHERLANDS

## Abstract

Reconstructing past sea levels can help constrain uncertainties surrounding the rate of change, magnitude, and impacts of the projected increase through the 21^st^ century. Of significance is the mid-Holocene relative sea-level highstand in tectonically stable and remote (*far-field)* locations from major ice sheets. The east coast of Australia provides an excellent arena in which to investigate changes in relative sea level during the Holocene. Considerable debate surrounds both the peak level and timing of the east coast highstand. The southeast Australian site of Bulli Beach provides the earliest evidence for the establishment of a highstand in the Southern Hemisphere, although questions have been raised about the pretreatment and type of material that was radiocarbon dated for the development of the regional sea-level curve. Here we undertake a detailed morpho- and chronostratigraphic study at Bulli Beach to better constrain the timing of the Holocene highstand in eastern Australia. In contrast to wood and charcoal samples that may provide anomalously old ages, probably due to inbuilt age, we find that short-lived terrestrial plant macrofossils provide a robust chronological framework. Bayesian modelling of the ages provide improved dating of the earliest evidence for a highstand at 6,880±50 cal BP, approximately a millennium later than previously reported. Our results from Bulli now closely align with other sea-level reconstructions along the east coast of Australia, and provide evidence for a synchronous relative sea-level highstand that extends from the Gulf of Carpentaria to Tasmania. Our refined age appears to be coincident with major ice mass loss from Northern Hemisphere and Antarctic ice sheets, supporting previous studies that suggest these may have played a role in the relative sea-level highstand. Further work is now needed to investigate the environmental impacts of regional sea levels, and refine the timing of the subsequent sea-level fall in the Holocene and its influence on coastal evolution.

## Introduction

Whilst global sea level has risen through the twentieth century and is expected to increase into the future, considerable uncertainties surround the timing, magnitude and impact of projected change [1, 2]. A major cause of this uncertainty is the short nature of the historic record and the limited range of observed changes compared to the recent geological past [3, 4], especially the response of ice sheets to warming [5–10]. The situation is exacerbated during the last century with sea-level rise being dominated by thermosteric effects [11]. Although the historic record can be extended back millennia by exploiting natural archives sensitive to sea-level change, including coastal sedimentary and geomorphological features [12–15], there is an urgent need for a greater network of sites in time and space [16]. This is particularly so given the non-linear nature of coastal inundation as a result of sea-level rise [17–19] and the major associated environmental and socio-economic impacts projected for the 21^st^ century [20–22].

Since the Last Glacial Maximum (LGM) at c. 21,000 years ago [23], the elevation of the world’s oceans has risen some 120 m to present mean sea level (PMSL) [24]. This rise in sea level has been nonlinear since the LGM [25–29], but with an average rate of ~1.2 cm/yr, which is comparable to projected rates of 21^st^ century sea-level rise [30, 31]. Globally, these changes had far-reaching impacts on both humans and ecosystems, particularly in Australia, which has an extensive network of archaeological and environmental records spanning the last 50,000 years [32–35]. Importantly, the spatial and temporal changes that occurred through the Holocene (the last 11,650 years) remain unclear in Australia [28, 31, 36, 37]. Potentially important is the mid-Holocene relative sea-level highstand (the period where relative sea level sustained the highest elevation above PMSL) where understanding this rise may provide an analogue for the future. A relative sea level highstand has been reported across Australasia and the wider Southern Hemisphere as well as north of the equator [36, 38–41]. Here we define the onset as representing the period from which relative sea-level rose above PMSL until the initiation of the highstand. Critically, previous work has argued the onset commenced between approximately 8,000 to 7,000 years ago [39, 42, 43], but reconstructions of sea-levels (using a variety of intertidal deposits including estuarine archives and sub-fossil mangroves) suggest significant spatial and temporal variability [38, 39, 44].

The origin of this early sea-level highstand remains unclear. Previous work has suggested that prolonged meltwater flux sustained the highstand through much of the Holocene [39, 45, 46]. The establishment of the Australian sea-level highstand at 8,000 years ago is surprising given the remote (*far-field*) location from major ice sheets [36]. Global ice sheets lost considerable mass after the LGM [23], with significant mid-Holocene ice-mass loss reported from the West and East Antarctic Ice Sheets [47, 48], and ongoing mass loss from Greenland throughout the Holocene [49, 50]. Given the sensitivity of ice sheets to greenhouse gas forcing [9, 51], the relationship to regional sea-level highstands needs to be better constrained particularly in the mid to low latitudes, that have been highlighted as key locations for potential future rapid sea-level rise [52].

Far-field sites that are tectonically quiescent, such as Australia, are of global significance as they are likely to preserve some of the most coherent records of ice-equivalent eustatic sea level [31, 36]. Whilst global mean sea level reflect multiple factors, the contribution made from the Earth's ice sheets, mountain glaciers and thermal expansion represent primary drivers that are modulated by other complex regional factors including ocean siphoning, continental levering, and climate influences [28, 43, 53]. Because of glacio-eustatic changes following the LGM, modelling studies suggest the eastern Australian coast has responded slowly to the reduction in global ice volume, and that sea-level reached its present level between 9,000 and 7,000 years ago [54], on which isostatic influences are superimposed [31, 46]. Despite extensive study, reconstructions from the Australian coast have struggled to reconcile the debate regarding the timing and elevation of a mid-Holocene sea-level highstand [39, 44]. For instance, in northeast Australia (Queensland) it is currently accepted that a highstand of 1 to 1.5 m above PMSL was reached around 7,000 years ago [31]. Conversely, in southeast Australia, early research suggested that sea-level reached present-day elevations 7,000 years ago and maintained PMSL through the mid- to late- Holocene [44, 55]. Subsequent work from Bulli Beach, a site ~80 km south of Sydney, however, has provided evidence for an early Holocene sea-level highstand of +1.84 m possibly dating back as early as 8,000 years ago [42, 56, 57]. The elevation and timing currently reported from Bulli Beach is anomalous, however, not just within Australia [39], but globally [36]. Importantly, virtually all of the data informing on this early Holocene highstand in Australia are from Bulli Beach and proximal sites [39].

Here we report on a detailed study revisiting the elevated estuarine sediments at Bulli Beach to better constrain the onset of the Holocene sea-level highstand and attempt to place these results in a global context.

## Previous studies

**Fig 1:**
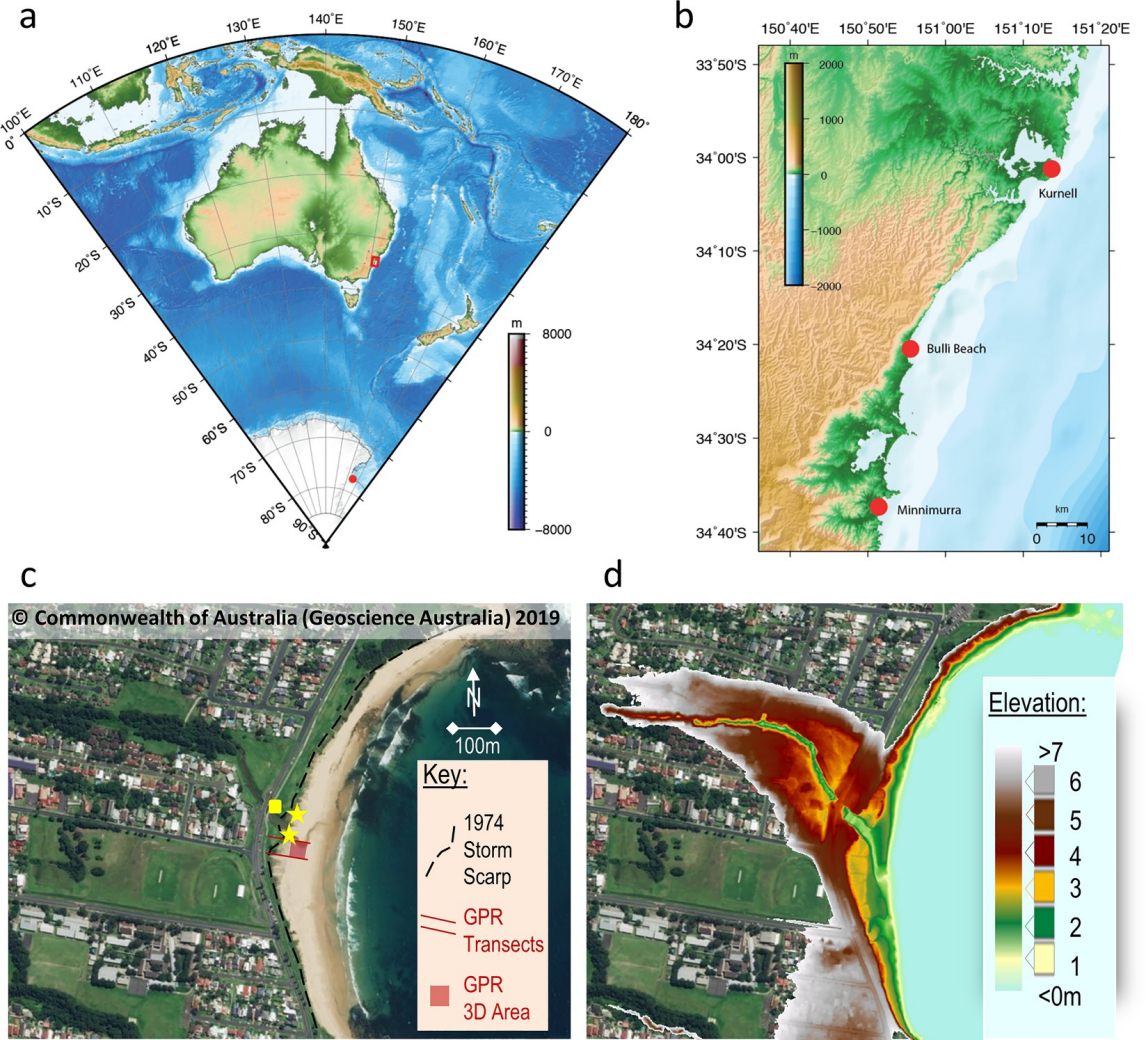
Location maps and images of Bulli Beach (New South Wales, Australia) showing key sites discussed in text. (a) Large scale regional map of Australia, (b) New South Wales coast with early Holocene Kurnell and Minnamurra sites shown in relation to Bulli (maps produced with GMT [98]). (c) Ariel image of Bulli Beach, overlain in (d) with a Digital Elevation Model (DEM) derived from LiDAR (figures based on material sourced from Geoscience Australia, 2019 [99]). Note, in panel c the yellow square shows the location of the bench mark, while the yellow stars denote ^14^C sampling sites north and south of Slacky Creek proximal to locations dated by Jones *et al*., 1979 [56]. The DEM highlights the extent of the receded barrier (maroon and brown) backing the central portion of Bulli Beach and bisected by Slacky Creek. Also note the beach (green) and low-lying dune (yellow and orange) that has built since the 1978 storm, burying the estuarine mud. While the aerial photograph (c) displays an accreted beach, the LiDAR (d) captures a post-storm recovery bar.

Bulli Beach, also known as Sandon Point Beach (hereafter ‘Bulli’), is approximately 900 m long with a varying width due to storm cut-and-fill (Fig 1). The modal beach state for Bulli grades between ‘transverse bar’ and ‘rip to low tide terrace’, with average wave heights ranging from 0.5 m in the south to 1 m in the north [58]. Bulli Beach is the seaward portion of a receded barrier complex that is covered with fill. The receded nature of this system is demonstrated by periodic erosion exposing an outcrop of grey, sandy estuarine mud in front of the barrier/fill and along the banks of Slacky Creek inlet. Typically following an erosive event, a post-storm recovery bar/ridge impounds Slacky Creek causing it to run parallel to shore and ultimately reburies the deposit (Fig 1C and 1D).

**Fig 2:**
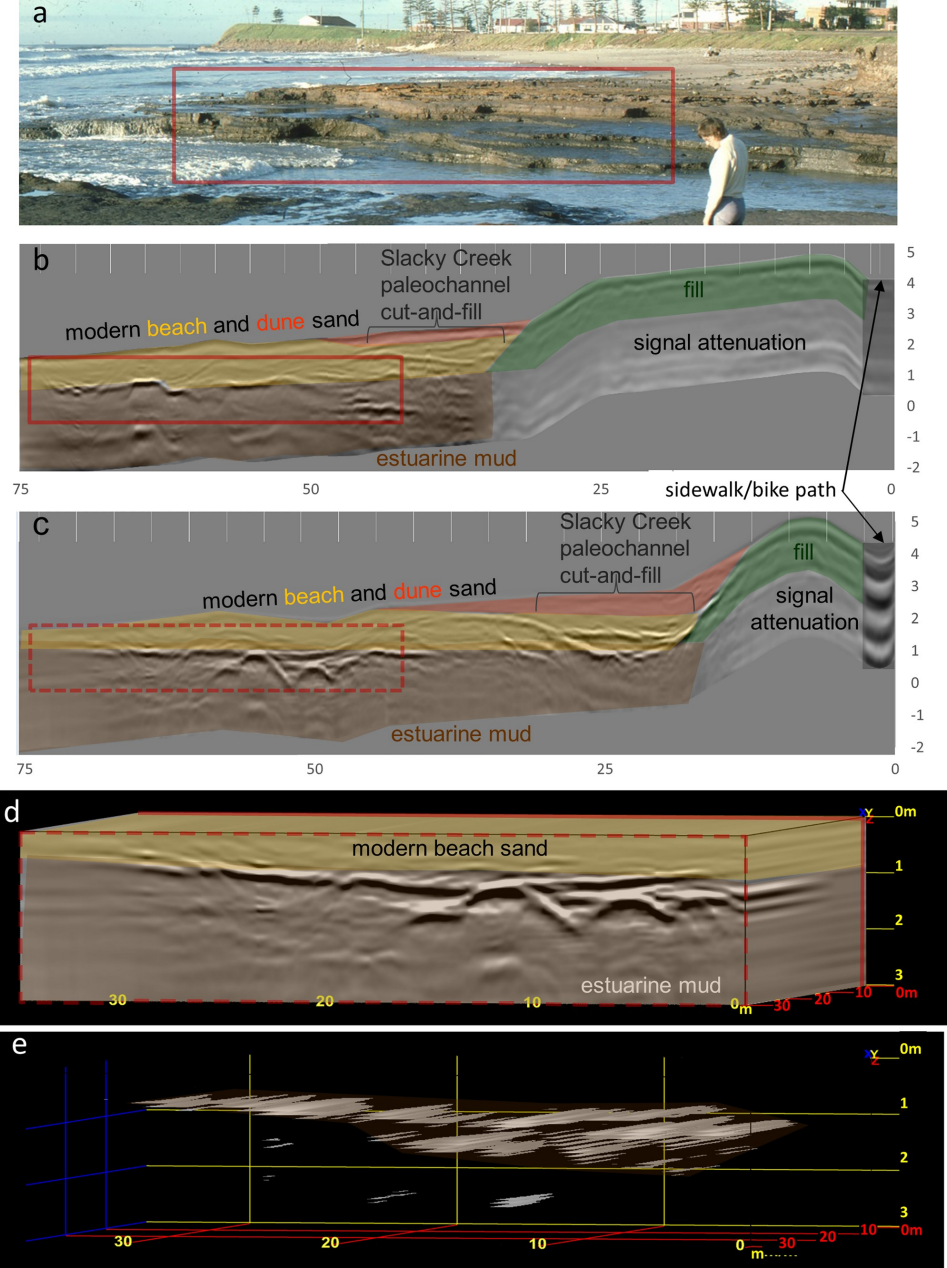
Photographs and GPR data of the estuarine mud deposit at Bulli Beach. (a) Oblique photograph looking south across Slacky Creek of estuarine mud exposed along Bulli beach after the 1978 storm (courtesy of Bob and Ann Young). (b) Northern GPR transect across present-day Bulli Beach imaging the buried estuarine mud surface with a strong reflection surface around an elevation of 1 m above MSL, similar to that found in the southern GPR transect (c). (d) The three-dimensional model used to image the estuarine mud surface in 3D (e). By isolating and interpolating between the high amplitude reflections caused by the peat surface, the lower amplitude signature within the overlying sand is stripped away, remotely sensing the lateral extent of the estuarine mud surface (e) previously exposed in 1978 (a).

A series of storms in the 1970s culminated in severe erosion at Bulli in 1978, exposing extensive early to mid-Holocene sedimentary deposits centered on Slacky Creek. Jones *et al*., 1979 [56] performed a comprehensive study of the Bulli barrier system using detailed coring and field mapping that capitalized on an extensive exposure of the usually buried back-barrier muds (Fig 2A) and identified four Quaternary units. The basal unit consists of Pleistocene fluvial muddy sands overlain by mottled estuarine mud sediments. The upper Holocene deposits are a grey, sandy estuarine mud capped by an upper sand unit representing the receded barrier that has been covered with fill to an elevation of 4 m above PMSL. Radiocarbon dating of wood, charcoal and shell material exposed within the estuarine mud at elevations between PMSL and +1.49 m appeared to suggest relative sea level had reached its present position between 7,500 and 6,400 BP [56]. This pioneering work also recognized a grey sandy mud unit at Thirroul (just north of Bulli) where ages obtained from charcoal (8,300±150 BP, equivalent to 9,210±190 cal BP) and a Myrtaceae root (7,000±150 BP, equivalent to 7,800±140 cal BP) were located 1.84 m above PMSL, although their provenance and association with Bulli was considered uncertain [56]. Note that the radiocarbon ages calibrated here used SHCal13 [59] and henceforth all calibrated radiocarbon ages are expressed as cal BP while uncalibrated ages are designated as BP. More than a decade after the benchmark study by Jones *et al*., 1979 [56], Bryant *et al*., 1992 [57] returned to Bulli and Thirroul as well as a neighboring site called McCauley’s Beach. Radiocarbon (^14^C) dating of shell and *in situ* mangrove stumps from estuarine deposits and thermoluminescence dating of quartz sand from beach deposits at elevations of 1–2 m above PMSL gave ages ranging between 6,900 BP and 1,520 BP respectively. The timing of this mid-Holocene highstand agrees with other results determined along the east coast of Australia and other far-field sites across the Southern Hemisphere [36, 39].

The above datasets have been utilized and combined with other records to construct regional sea-level curves for southeast Australia [39, 42]. Notably, the ages reported by Jones *et al*., 1979 [56] and Bryant *et al*., 1992 [57] form all the early data points above PMSL (>7,000 years ago) that have been used to mark the onset of mid-Holocene highstand, approximately a millennium prior to that elsewhere [36, 42]. While these more recent studies have recalibrated the older ages, and provided vertical error uncertainties to the sea-level reconstruction, these sites have not been revisited nor had new data been collected.

## Methods

Here we report an extensive morpho- and chronographic study of this important area using aerial photographs, Light Detecting and Ranging (LiDAR), ground penetrating radar (GPR), augers/cores and radiocarbon (^14^C) dating. Since none of the material from the original studies was available to redate, the field campaign aimed to recollect similar samples from the same depositional environments previously dated. Reconnaissance on Bulli, Thirroul and McCauley’s Beach yielded no evidence of the deposits previously dated [56, 57]. Since the deposits dated at Bulli Beach were the only ones with detailed location data available, they became the focus of the study. The aerial photographs and LiDAR images of the present-day barrier and beach at Bulli show how the estuarine deposits studied by Jones *et al*., 1979 [56] are not typically visible. To detect and map the lateral extent of the estuarine deposits exposed during the 1970s storms but buried by modern beach and dune processes, GPR was used to image the shallow subsurface. Augers used to ground-truth the GPR did not penetrate the estuarine mud. Coring through the modern beach and dune sands was subsequently undertaken to sample these deposits. Shortly after coring, a major storm in June 2016 re-exposed the estuarine deposits allowing surface and outcrop sampling at Bulli and McCauley’s Beach.

### Ground penetrating radar

GPR transects were collected perpendicular to the shoreline at Bulli in the central portion of the barrier (Figs 1 and 2). The GPR was collected in a grid configuration in order to produce a 3D image of the buried estuarine surface [60]. The GPR transects and associated beach profiles were topographically surveyed using a dumpy level. A SIR-3000 digital GPR system with a 200 MHz antenna from GSSI (Geophysical Survey System Inc., USA) was used to acquire the geophysical records. Processing (topographic corrections, normalization, stacking and depth conversions) and analyses were performed on unfiltered data using RADAN7 Software and 3D Module. Unfiltered data were used in the analysis, because GPR records are subject to noise at a range of frequencies, and only modest improvements were attained in radar stratigraphy following the use of a Finite Impulse Response filter, as well as filtering of phantom hyperbola and minor antenna ringing [40]. Gain adjustments were made in both processing and presentation of some records to increase the signal amplitude and the display resolution of stratigraphy. Travel-time was converted to depth in RADAN based on estimated dielectric constants [61] and ground-truthed using a hand auger. Sediments recovered from the auger were analyzed using standard sedimentological techniques for grain-size, sorting, rounding, and composition for comparison to the geophysical record of barrier facies. The interpretations of the recorded facies are described following the terminology of van Heteren *et al*., 1998 [61] and facies interpretations are guided by Jones *et al*., 1979 [56].

### Sampling

**Fig 3:**
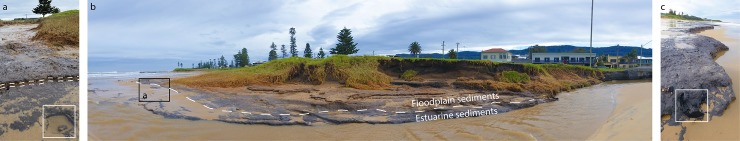
Photographs of the exposed sediments sampled for this study at Bulli Beach (taken shortly after the June 2016 storm). (a) Core location (white box) with limonite deposit marking the upper boundary of the estuarine sediments (white dashed lines). (b) Panorama of south-facing exposed estuarine sediment deposits (boundary with floodplain sediments marked by white dashed line). (c) North-facing view of the exposed estuarine sedimentary unit, with the dated wood (white box). Note, the keys used for scale in panels a and c.

On the south side of Slacky Creek at Bulli Beach (34.335°S, 150.925°E) coring of the estuarine sedimentary deposit below the upper sand unit defined by Jones *et al*., 1979 [56] was undertaken using a modified 5-cm diameter Livingstone corer. The corer was drilled through the estuarine sediments down to a level of -0.15 m PMSL. We were not able to penetrate the base of the estuarine sediments. The exposed section was surveyed using a dumpy level across the site, with altitudes relative to PMSL/AHD (Australian Height Datum, the official height datum for the country and equates to mean sea level). This was grounded to a survey datum based on the bridge immediately inland of the site (as used by Jones *et al*. 1979), which was resurveyed for this study using differential GPS. Following the storm of June 2016, extensive deposits of estuarine sediments were exposed on both sides of Slacky Creek. On the north side (34.334°S, 150.925°E) large wood fragments, including a *Melaleuca* log, were identified in the upper part of the deposit (Fig 3). Surface samples of wood were collected from the exposure (including two contiguous blocks of wood from the outer part of the *Melaleuca* log) for ^14^C dating. No surface deposit with an elevation of +1.49 m was identified (Fig 3C) [56]. During repeated visits north of Bulli Beach to Thirroul (2.5 km north of Bulli), we were unable to locate the exposure from which the highest point of the Holocene highstand had been reported [56]. However, following the June 2016 storm, exposures of sloping mottled estuarine sediments were exposed 1.2 km north of Bulli at 0.6 m PMSL, McCauley’s Beach (34.324°S, 150.925°E) [57]. Surface samples of degraded wood were collected for ^14^C dating. The sites were not protected and required no specific permissions for sampling. No endangered or protected species were located at or near the sampling locations.

### Radiocarbon dating and age modelling

To collect a series of stratigraphically-constrained ages from Bulli Beach, the cores from the south side of Slacky Creek were extracted in the laboratory and subsamples were selected, soaked in Milli-Q^TM^ grade water and sieved through a 100 micron sieve. Short-lived terrestrial plant macrofossils, comprising fruits and leaves, formed the focus of our study, being fragile and less likely to be integrated intact into the sediments if remobilized [62]. For ^14^C dating, these samples were given an acid-base-acid (ABA) pretreatment, comprising 1N HCl at 70°C, rinsed and treated with multiple hot (70°C) 1N NaOH washes. The NaOH insoluble fraction was treated with 1N HCl at 70°C, filtered, rinsed and dried. Because some of the oldest ages previously reported for the highstand were reported from bulk wood (e.g. an *in situ* stump from +1.09 m of 6,890±220 BP reported by Bryant *et al*., 1992), we undertook alpha-cellulose extraction of this material type. Cellulose is considered the most inert component of wood, making it ideal for radiocarbon dating [63]. Methods used for the extraction of holocellulose do not always remove all lignin and residual contaminants, requiring a further alkali extraction to produce alpha-cellulose that more directly reflects atmospheric ^14^C levels during photosynthesis [64, 65]. Chemical pre-treatment of the wood samples resulted in the purification of alpha-cellulose as this wood fraction is deemed the most reliable for minimizing potential contamination and providing the most robust ^14^C ages required for such high-precision study [64]. Alpha-cellulose extraction begins with an ABA pretreatment at 80°C, with samples treated with 1N HCl for 60 min, followed by successive 30 min treatments with 1N NaOH until the supernatant liquid remained clear, ending with another 60 min 1N HCl wash. Holocellulose was then extracted by using successive 30 min treatments of acidified NaClO_2_ at 70°C until the wood shavings were bleached to a pale-yellow color. Alpha-cellulose was then prepared by a final treatment with NaOH followed by a further acid wash (1N HCl at 70°C for 30 min), and repeated washing with distilled water until a pH of >6 was achieved. Samples were combusted and graphitized in the Waikato Radiocarbon Laboratory and ^14^C/^12^C measured by Accelerator Mass Spectrometry (AMS) at the University of California at Irvine (UCI).

**Fig 4:**
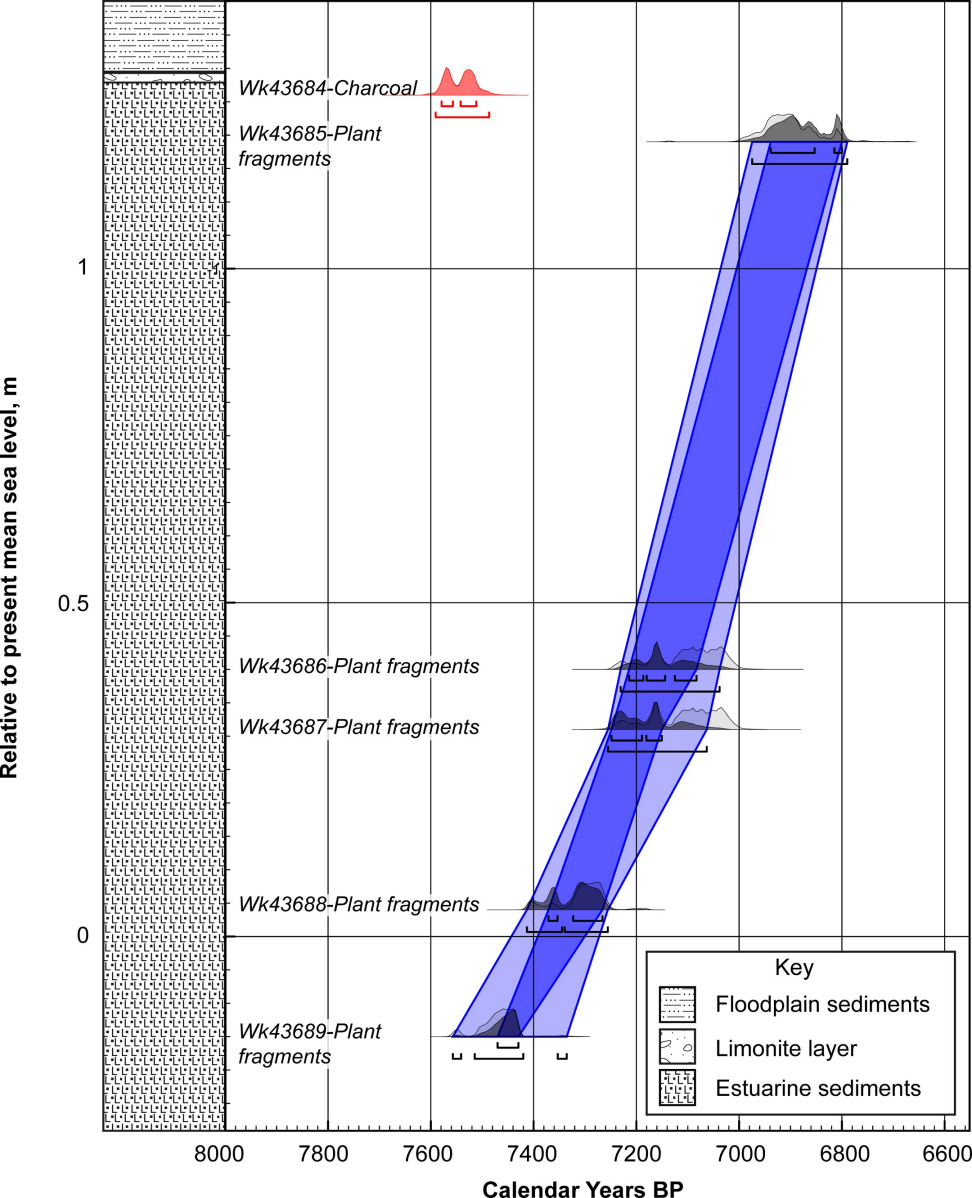
Lithostratigraphy and OxCal age-depth model for the Bulli Beach (Slacky Creek South, NSW) using SHCal13 [59]. The posterior and prior probability distributions are shown as dark and light respectively. The dark and light blue envelope provide the 1σ and 2σ calibrated age range respectively. Radiocarbon age laboratory numbers are denoted by the prefix ‘Wk’. The anomalously older charcoal sample is shown in red.

To help to constrain the timing of the highstand, Bayesian age modelling exploits the stratigraphic ordering of the radiocarbon ages [66]. This ‘prior’ information helps to reduce the uncertainties associated with calibrating radiocarbon ages and improve the age control. The ^14^C ages from the stratigraphic sequence at the Slacky Creek (south) site were used to develop an age model using a P_sequence deposition model in OxCal 4.2 [67, 68] with the General Outlier analysis detection method (probability = 0.05) [69]. The ^14^C ages were calibrated against the Southern Hemisphere calibration (SHCal13) dataset [59]. The model was based on 30,000 iterations. Using Bayes’ theorem, the algorithms employed sample possible solutions with a probability that is the product of the prior and likelihood probabilities [67]. Taking into account the deposition model and the actual age measurements, the posterior probability densities quantify the most likely age distributions; the outlier option was used to detect ages that fall outside the calibration model for each group, and if necessary, down-weight their contribution to the final age estimates (Table 1 and Fig 4). Only ages that are considered contemporaneous with sedimentation have been calibrated.

**Table 1:** Radiocarbon ages for Bulli and McCauley’s beach estuarine sediments. The Bulli ages (obtained on the south side of Slacky Creek) have been modelled using the P_sequence and Outlier analysis option in OxCal 4.2 [100, 101] with SHCal13 [59]. A_model_ = 91.2; A_overall_ = 83.

Profile and height above PSL, m	Wk lab number	Material	^14^C BP ± 1 σ	Modelled mean cal age (years, BP ± 1σ)	Modelled mean cal age (years, BP ± 2σ)
*Slacky Creek (south)*					
1.26	*43684*	Charcoal	6720±20		
1.19	*43685*	Fruits and leaves (unidentified)	6100±20	**6880±50**	**6900±100**
0.40	*43686*	Fruits and leaves (unidentified)	6250±20	**7140±50**	**7100±100**
0.31	*43687*	Fruits and leaves (unidentified)	6250±20	**7170±50**	**7200±100**
0.04	*43688*	Fruits and leaves (unidentified)	6410±20	**7320±40**	**7320±90**
-0.15	*43689*	Fruits and leaves (unidentified)	6610±20	**7450±30**	**7450±60**
*Slacky Creek (north)*					
1.27	*43819*	Degraded wood fragments (unidentified)	6530±20		
1.27	*43820*	Degraded wood fragments (unidentified)	6380±20		
1.13	*43821*	Outer edge of *Melaleuca* log	6210±20		
1.13	*43822*	Contiguous (inner) sample of *Melaleuca* log	6300±20		
*McCauley’s Beach*					
0.6	*43923*	*Dicksonia antarctica* fragments	6820±20		
0.6	*43924*	*Dicksonia antarctica* fragments	6610±20		

## Results and discussion

### Evidence for a sea-level highstand at Bulli

The GPR data collected at Bulli clearly imaged the estuarine muds from Jones e*t al*., 1979 [56] (Fig 2B and 2C). The top of this facies is well defined by a strong, flat to stepped reflection that is laterally extensive at ~1 m above PMSL (Fig 2B–2E) similar to that exposed in the 1978 storm (Fig 2A). The overlying beach facies consists of medium- to coarse-grained quartz sand that produces mid-strength reflections between ~1–2 m above PMSL. Stratigraphy in the landward beach facies displays the post-storm recovery after 1978 (Fig 2B and 2C). The distinct ridge and runnel stratigraphy formed as eroded sands migrated back onshore in the form of a bar (ridge), which created a landward swale (runnel). Slacky Creek likely ran through the runnel until the channel cut became pinned along the barrier and subsequently filled during berm formation. This process has been observed after smaller storms that did not erode back to the 1978 scarp (e.g. Fig 1C and 1D). The dune facies overlying these berms are expressed by a thin reflection-free signal in the central portion of the GPR record to a depth of ~2 m above PMSL (Fig 2B–2D). This homogenous geophysical signature is representative of the well-sorted, fine-grained, quartz-rich sand, transported by aeolian processes. Where the berm is wider in the south, incipient dune vegetation has colonized the relatively flat-lying aeolian veneer (Fig 2C).

Within the attenuated signal beneath the fill there is a layer preserved above the estuarine mud that was not imaged in the GPR but was recorded by outcrop mapping (Fig 3). The base of this facies on the south side of Slacky Creek between 1.26–1.28 m above PMSL comprises a coarse-grained limonite layer described as the base of the upper sand unit recognized by Jones *et al*., 1979 [56]. Importantly, limonite represents a mixture of similar hydrated iron oxide minerals, formed as a result of oxidation in water-rich sediment [70], and commonly found within marshy sediments [71]. The presence of limonite is consistent with the establishment of floodplain sediments at elevated sea-level (Fig 3). We found no evidence of the estuarine muds at elevations of +1.49 to +1.84 m above AHD at any of the sites.

While no mangrove stump at +1.05 m was found [56], all of the samples collected for this study are from a proximal location within the same estuarine unit (Fig 1). It was also possible to collect surface samples of wood and charcoal at similar locations and elevations as those analyzed in Jones *et al*., 1979 [56]. A continuous core was collected from +1.19 m down to –0.15 m AHD. These intertidal and mangrove sediments were used to indicate when sea-level reached a particular height, as previously done in Australia and globally [38, 72–74].

The indicative meaning (IM), indicative range (IR), reference water level (RWL), and associated errors for these sea-level index points were calculated using established protocols and formulas [75, 76]. The IM is comprised of the IR (which is the range over which the sample types occurs in the modern environment) and RWL (which is usually the mid-point of the IR). Due to the highly urbanised nature of the study site and the limited accommodation space to support mangrove, the current vertical range of mangrove within the tidal frame at this location could not be determined. Data extrapolated from nearby estuaries indicate that the mean elevation of mangroves is approximately 0.36 m extending to lower elevations of 0 m and upper elevations of up to 1 m AHD [77]. The upper 75% quantile indicates that most mangrove are positioned below 0.52 m AHD. This elevation corresponds to the mean high water mark (0.5 m AHD) measured at the Fort Denison, Sydney, tide gauge [78]. Mangrove roots also extend to elevations below the surface to a maximum depth of approximately 50 cm [79]; which when applied to the lower elevation of mangrove distribution corresponds to the mean low water mark (-0.51 m AHD) at Fort Denison. Based on this the IM is mean high water (MHW) to mean low water (MLW), with an IR for mangrove organic material accumulation of -0.5 to 0.5 m AHD and a RWL of 0 m AHD. This is consistent with estimates elsewhere in the region that use an IR between mean high water and mean low water [38, 39, 42], and presumes that the current tidal range is consistent with palaeotidal range at the time the organic material accumulated. The vertical range of mangrove was defined using a Light Detection and Ranging (LiDAR)—derived digital elevation model with reported accuracy of 15 cm, which was validated using an RTK-GPS with similar accuracy; the tidal plane analysis has a reported error of up to 0.034 m and the RTK-GPS used to define the vertical elevation of the core had an accuracy of 15 cm. These values were used to generate a vertical error estimate of 0.21 m, which excludes the effects of sediment consolidation on IM.

### Dating the onset of the Holocene relative sea-level highstand

Previous work on the Holocene highstand at Bulli and the wider area has highlighted the offset between ages obtained from different materials (for example, wood and charcoal) and components (cellulose and bulk) [56, 57]. These differences have been widely reported from other contexts [80–82], raising the possibility that the early highstand of ~8 ka cal BP [36, 39] may be related to pretreatment and/or reworking of older material, rather than reflecting a true event. To investigate these issues, we undertook a comprehensive dating program of wood, charcoal and short-lived plant macrofossils (Table 1).

The short-lived terrestrial plant macrofossils provide a robust sequence of ages from the south side of Slacky Creek indicating accumulation began at 6,610±20 BP (-0.15 m PMSL; Wk-43689) to 6,100±20 BP (+1.19 m PMSL; Wk-43685) (Table 1). Bayesian age modelling of the series suggests the sediments represent a period spanning 7,450±30 cal BP to 6,880±50 cal BP (Fig 4). In contrast, a charcoal sample taken immediately underlying the limonite layer and representing the highest point of the estuarine sediments in our sequence (+1.26 m PMSL), reported a radiocarbon date of 6,720±20 BP (Wk-43684), significantly older than the ages obtained from the short-lived macrofossils dated below it (Fig 4). Unfortunately, no terrestrial macrofossils were identified in this uppermost sample. Importantly, radiocarbon dated surface wood on the north side of Slacky Creek at comparable heights to the uppermost sediments on the south side (+1.27 m PMSL), reported similar older ages of 6,530±20 BP (Wk-43819) and 6380±20 BP (Wk-43820) (Table 1). Similarly, at a relatively lower elevation, the ages obtained from McCauley’s Beach at +0.6 m PMSL were also relatively older compared to the Bulli Beach (south Slacky Creek series), with the oldest age obtained being 6,820±20 BP (Wk-43923).

**Fig 5:**
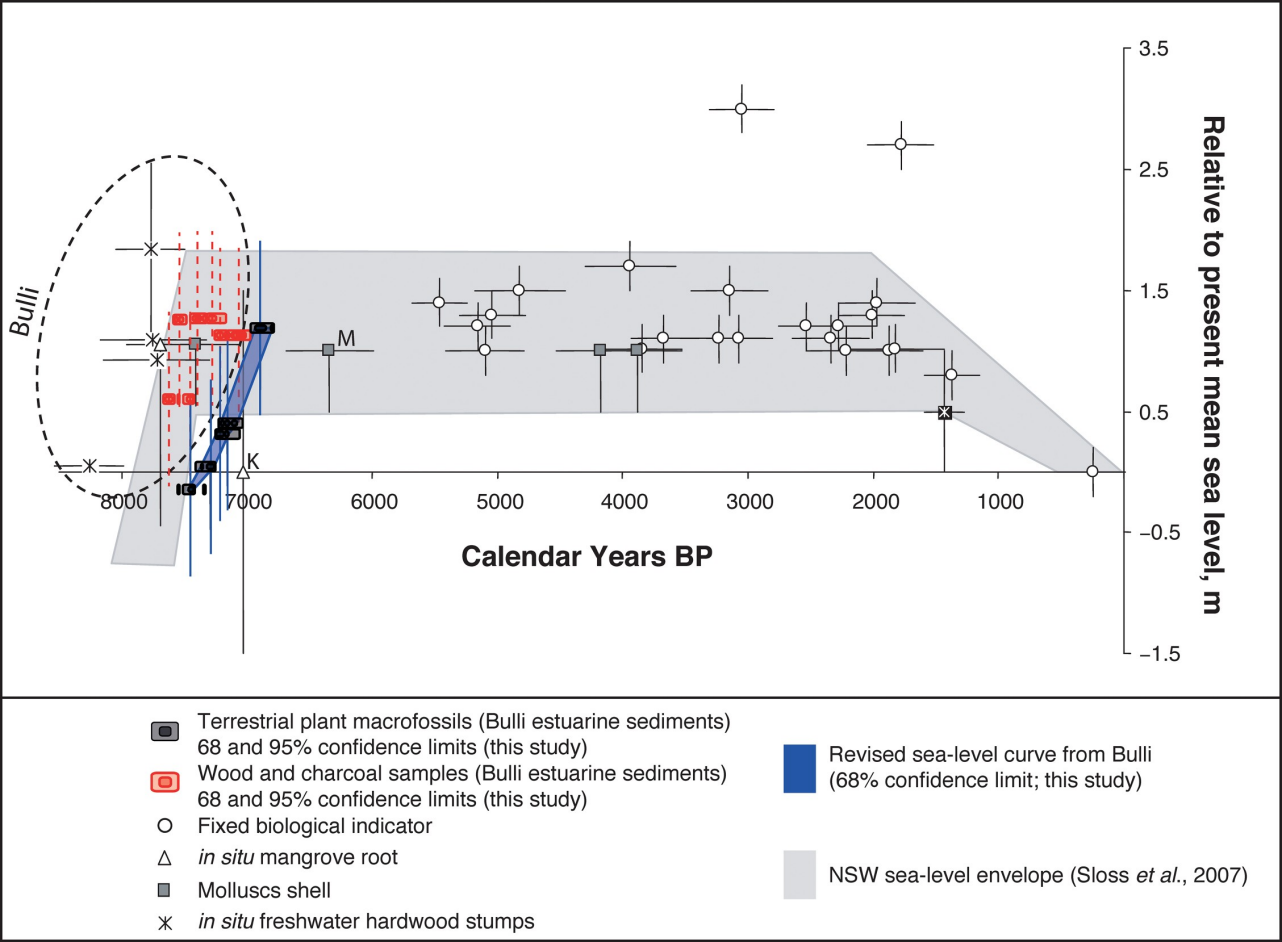
Comparison between Bulli Beach (Slacky Creek; this study in red and blue) with the previously published Holocene sea-level curve for New South Wales in greyscale [42]. All of the oldest dates on the previously published sea-level curve are from Bulli (denoted by dashed circle), with the next two oldest dates from nearby sites labeled K for Kurnell and M for Minnamurra (see Fig 1 for locations). Anomalously older wood and charcoal samples from this study are plotted for comparison (in red).

Our results are consistent with reworking and subsequent re-incorporation of wood and charcoal into the estuarine sediments, suggesting these are not reliable material types for dating the timing of sea-level change (at least at Bulli and the immediate area). Whilst charcoal and wood are typically more durable than short-lived components (such as leaves, fruits and seeds) they suffer from an inbuilt age, relating to the time between when the carbon is fixed within the structure of the plant and its subsequent death [83]; depending on the lifespan of the plant, the inbuilt age can range from decades to centuries (or in extreme situations, millennia) [84, 85], and may contribute to the discrepancies observed. More importantly, however, is that Bayesian age modelling helps refine the timing of sea-level rise in the approach to the highstand. In contrast to other studies investigating datasets comprising individual dated site locations [39, 42], Bayesian age modelling is able to exploit a stratigraphically-constrained sequence of ages [68, 69], providing additional chronological control. Our findings indicate that the highstand represented by the remaining sediments at Bulli was reached at 6,880±50 cal BP, approximately one millennium later than previously reported for these deposits [39, 42, 56] (Fig 5).

### Wider implications

The refined age modelling of short-lived plant macrofossils provides a coherent rise in relative sea level from PMSL at 7,500 cal BP to a highstand of +1.19 m at 6,900 cal BP (Fig 5). Our Bayesian age modelled suite of radiocarbon ages provide a coherent chronological framework for Bulli Beach (southeast Australia) and suggest the onset of the mid-Holocene sea-level highstand occurred approximately a millennium later than previously reported at this site [39, 42, 56]. Importantly, our new results agree with other records from the Sydney region. Roy and Crawford, 1976 [86] obtained a comparatively young ^14^C age from Kurnell Peninsula at PMSL of 6,220±115 BP (7,070±140 cal. years BP) from a fossil mangrove stump (*Avicennia marina*), which in contrast to the original interpretation, more likely reflects an early part of the Holocene rise and not a stabilization of sea-level (Figs 1 and 5). An oyster shell from Minnamurra, ~40 km south of Bulli [42] (Fig 1) recorded an age of 5,950±120 BP (6,380±150 years cal BP; calculated using Marine13 and a ΔR value of 3±69 [87, 88]) at +1 m PMSL is also consistent with our findings (Fig 5).

We therefore conclude that irrespective of the pretreatment method used, the relatively old reported ages from Bulli and surrounding environments that have been used to generate a regional sea-level curve appear to be the product of reworked material (Fig 5). Crucially, our results suggest a coherent picture of synchronous relative sea level reaching or exceeding PMSL along the east coast of Australia [39] extending from the Gulf of Carpentaria to Tasmania [14, 38, 89, 90]. Taken together our results support the initiation of a continental-wide sea-level highstand shortly after 7,000 cal BP.

The onset of the Holocene highstand had critical impacts on hunter-gatherer societies across the globe. International studies have implicated sea-level change during this period as the stimulus for significant societal change, including at least in part the process of neolithisation of the Mediterranean and the subsequent spread of agriculture across Europe [27, 91, 92], and the submergence of Doggerland, resulting in the differing demographic and socio-economic development of Britain and mainland Europe [93–95]. In Australia, these impacts were likely far greater, with populations only just recovering from displacement and adjustments stemming from the rapid inundation of the continental shelf during the terminal Pleistocene [37, 89, 96]. Specifically, a recent study has shown that between ~15–8 ka cal BP, and associated with Meltwater Pulse 1a, the continent lost some 2 million km^2^ (about 20% of its total landmass) at a maximum pace of coastal retreat of ~23.7 m per year [37]. This rate of innundation would have resulted in significant disruption of coastal productivity and resources that underpinned coastal foraging economies in the late Holocene, as well as further reducing the spatial area within which populations could move and occupy. These impacts would likely have required ongoing changes in mobility, technology and behavior. Previous studies have assigned numerous Holocene technological and behavioral changes (e.g. diversification of archaeological sites, microlithisation of stone artefacts, expansion of Pama-Nyungan language) to ameliorating climate [33]. These changes instead may partly reflect increased density of populations along the eastern seaboard. These areas still contain some of the densest populations of the continent. Archaeologically, it is important to highlight that the highstand would have resulted in the modification and/or loss of any coastal sites that would have formed between the LGM and ~6,900 cal years BP. One consequence of which is that researchers focusing on this time period need to carefully consider their interpretations for taphonomic bias.

Importantly, the revised ages from eastern Australia now align with other sites around the country and are consistent with global studies [14, 31, 36, 38, 89]. Furthermore, our results are broadly similar with the timing of major mass loss from the West and East Antarctic Ice Sheets from 6,800 cal BP [47, 48] and ongoing mass loss from Greenland throughout the Holocene [49, 50] (Fig 5). Previous work has suggested that these ice-sheet mass losses may have sustained the elevated sea-level along the east coast of Australia [39, 45, 97]. Our findings support this proposal, while recognizing the contributions of continental levering and ocean siphoning [46, 53, 54]. Future work is needed to more precisely constrain the timing and impact of Holocene ice mass loss and contribution to regional/global sea-level rise.

### Conclusions

Reconstructing past sea level can help constrain uncertainties surrounding the rate of change, magnitude and impacts of projected increases through the 21^st^ century. Of significance is the mid-Holocene sea-level highstand (+1 m PMSL) which potentially provides an analogue for 21^st^ century warming projected to rise to similar elevations. In Australia, considerable debate surrounds the existence and timing of a mid-Holocene highstand, which has hitherto been considered spatially and temporally complex. Crucially, the area known as Bulli Beach in southeast Australia provides the earliest evidence for the establishment of a highstand in the Southern Hemisphere. However, the initial studies have been critiqued, notably in relation to sample pretreatment and material type.

Here, we revisit Bulli Beach and undertake a detailed morpho- and chronostratigraphic study. We find that regardless of the pretreatment method used, wood and charcoal samples provide anomalously old ages, probably the result of in-built age. Instead, we targeted short-lived terrestrial plant macrofossils that more accurately reflects the timing of sea-level change. Bayesian age modelling of stratigraphically-constrained series of ages from these types of samples provides a method for reducing the envelope of uncertainty of sea-level rise, and suggests the initiation of the mid-Holocene highstand was approximately one millennium later than previously thought at 6,880±50 cal BP. Our results are consistent with other records from across Australia and globally. Further work will refine the structure of the sea-level highstand and the timing of sea-level fall through the late Holocene.
